# Effects of Recreational Physical Activity on Abdominal Obesity in Obese South Korean Adults

**DOI:** 10.3390/ijerph192214634

**Published:** 2022-11-08

**Authors:** Yoonmi Lee, Sungjung Kwak, Jieun Shin

**Affiliations:** 1 Department of Health Exercise Management, Sungshin Women’s University, Seoul 02844, Republic of Korea; 2 Robotic Surgery Center, Konyang University Hospital, Daejeon 35365, Republic of Korea; 3 Department of Biomedical Informatics, College of Medicine, Konyang University, Daejeon 35365, Republic of Korea

**Keywords:** abdominal obesity, recreational physical activity, Korea National Health and Nutrition Examination Survey (KNHANES)

## Abstract

This study investigated the effects of general characteristics, health behaviors, and level of physical activity on abdominal obesity in obese adults (BMI (body mass index) ≥ 25 kg/m^2^) using data from the seventh period (2016–2018) of the Korea National Health and Nutrition Examination Survey (KNHANES). We also prepared basic data on the improvement and management of abdominal obesity. The participants were 2343 obese adults (men, 1338; women, 1005) from the KNHANES. Factors relevant to abdominal obesity in obese Korean women were general characteristics (age, marital status, occupation, education, and menopause) and health behaviors (time for recreational physical activities and energy intake). In men, these factors tended to be health behaviors, including time spent on leisure physical activity, and sitting. It was found that over 600 Mets/week of recreational physical activity for both adult men and women reduced the incidence of abdominal obesity after adjusting for general characteristics and health behaviors (odds ratio (95% CI); men 0.69 (0.51–0.92); women, 0.61 (0.40–0.94)). Therefore, to prevent or improve abdominal obesity in obese adults in Korea, it is necessary to consider general characteristics and health behaviors according to sex. In addition, maintaining a physical activity rate of over 600 Mets/week is also recommended.

## 1. Introduction

Since 1975, the prevalence of obesity has increased more than threefold worldwide [1]. Obesity was also linked to 8% of global deaths in 2017 [2]. Therefore, obesity has gained international attention as an important public health concern. In the United States, the prevalence of obesity increased from 13.4% in 1960 to 30.9% in 2000 [3] and 42.4% in 2017 [4]. In South Korea, the prevalence of obesity in adults increased from 29.2% in 2001 to 33.8% in 2019 [5]. Although the increase in the obesity rate in South Korea was less than that in the United States, one in three South Korean adults is obese, further highlighting obesity as an important social issue.

Obesity is associated with elevated free fatty acid levels. Subsequently, increased insulin resistance may cause cardiovascular diseases and other conditions, such as diabetes mellitus, dyslipidemia, and hypertension, further increasing the risk of target organ damage, such as kidney disease [6,7,8]. Abdominal obesity has been identified as an essential contributor to the development and progression of metabolic syndrome (MetS) by the Adult Treatment Panel (ATP) III of the National Cholesterol Education Program (NCEP), International Diabetes Federation (IDF), and World Health Organization (WHO) [9,10,11]. Waist circumference (WC) is a commonly used indicator of increased abdominal fat. Studies have reported that WC is a more accurate predictor of mortality caused by cardiovascular disease than body mass index (BMI) [12,13]. As reported in previous studies, obesity and waist circumference increase the risk of cardiovascular disease, so efforts need to be made to improve them.

Recommendations for preventing overweight and obesity include consuming a healthy diet, which involves limiting energy intake from total fat and sugar and exercising for 60 min per day for children or 150 min per week for adults [1]. In addition, the physical activity (PA) guideline from the American College of Sports Medicine (ACSM) recommends moderate-intensity PA of 150 min/week and high-intensity PA of 75 min/week to improve and maintain health in adults [14,15]. However, some studies report that 250–300 min/week [16,17] or 300–400 min/week [18] of aerobic exercise is required for weight and fat reduction, so it is necessary to consider the difference in exercise time according to obesity status. In addition, it is reported that the rate of aerobic exercise among Korean adults is continuously decreasing [19], but studies examining the characteristics of obese people according to the level of physical activity practice are insufficient.

Therefore, this study used Korea National Health and Nutrition Examination Survey(KNHANES) VII (2016–2018) data to examine the impact of the general characteristics, health behaviors, and PA of obese adults (BMI ≥ 25 kg/m^2^) on abdominal obesity and to provide basic data for the improvement and management of abdominal obesity.

## 2. Materials and Methods

### 2.1. Design

This study is a secondary data analysis study that identified various levels of regular PA and obesity-related factors in South Korean adults using data from the 7th KNHANES (2016–2018). This study was described in accordance with the Strengthening the Reporting of Observational Studies in Epidemiology (STROBE) guidelines (https://www.strobe-statement.org/index.php?id=strobe-home (accessed on 4 November 2022)).

### 2.2. Participants

Of the 24,269 individuals included in the 7th Korea National Health and Nutrition Examination Survey (KNHANES VII) (2016–2018), 4661 satisfied the following inclusion criteria: ≥19 years old and a BMI (body mass index) ≥ 25 kg/m^2^. The researchers excluded respondents according to the following criteria: participants who answered “no” to the question of whether they were physically active (PA at work, during recreation, or transportation) (*n* = 258), diagnosed with cancer (*n* = 142), pregnant or breastfeeding (*n* = 52), diet modification (*n* = 208), <50 kcal or ≥500 kcal energy intake (*n* = 823); and individuals with a limitation in PA (*n* = 242). Therefore, 2343 participants (1338 men and 1005 women) were included in this study.

### 2.3. Materials

#### 2.3.1. Assessment of Obesity

BMI [weight (kg)/height (m)^2^] was used to classify obese adults. According to the WHO obesity guidelines for Asian populations, obesity is defined as BMI ≥ 25 kg/m^2^ [20]. Furthermore, according to the Korean Society for the Study of Obesity guidelines, abdominal obesity is defined as WC ≥ 90 cm in men and ≥85 cm in women [21].

#### 2.3.2. Classification of Health Behavior

Health behavior was determined using the following factors: smoking, drinking, hours of sleep, level of energy intake, and level of PA. Participants were categorized into the following groups based on their smoking status: “current smoker” for current smokers who reportedly smoked cigarettes daily or occasionally, “former smoker” for those who were not current smokers but had a history of smoking, and “never smoker” for those who had never smoked. Regarding drinking, “non-drinker” included participants who never drank alcohol or drank less than once a month within the last 12 months, while “drinker” was used for those who drank ≥ once a month within the previous 12 months. Hours of sleep were categorized into “<7 h,” “7–8 h,” and “≥8 h.” Regarding the level of energy intake, the 2015 Dietary Reference Intakes for Koreans was used as a reference. When the participants’ intake was greater than the estimated energy requirement (EER) for their sex and age group, their energy intake was indicated as “adequate.” In contrast, their intake was indicated as “inadequate” when it was below the EER [22]. The Global Physical Activity Questionnaire (GPAQ) was used to determine PA levels and categorize them into “activity at work,” “recreational physical activities,” and “travel to and from places.” Questions related to physical activities included: “Do you do any vigorous or moderate-intensity activities that cause a large or small increase in breathing or heart rate for at least 10 min continuously?”, “How much time do you spend performing vigorous or moderate-intensity activities on a typical day?”, “In a typical week, on how many days do you perform vigorous or moderate-intensity activities?” The hours of exercise per week (wk) were calculated, and PAs were categorized based on intensity level into moderate-intensity exercise (150 min/week × 4 METs) and high-intensity exercise (75 min/week × 8 METs). In the classification of physical activity, those who answered “no” to the physical activity question were given “NO,” whereas others were classified as “<600 Mets/week” or “≥600 Mets/week,” depending on their level of PA. In addition, questions about travel to and from places included, “Do you walk or use a bicycle (pedal cycle) for at least 10 min continuously to get to and from places?”, “In a typical week, how many days do you walk or bicycle for at least 10 min continuously to get to and from places? ”, “How much time do you spend walking or bicycling for travel on a typical day?”, “Sitting time refers to sitting or reclining at work or home, getting to and from places, or spending time with friends, including sitting at a desk, sitting with friends, traveling in a car, bus, or train, reading, playing cards, or watching television. However, this did not include the time spent sleeping. The time spent sitting per day is presented in minutes after excluding the time spent sleeping [23,24].

### 2.4. Data Analysis

SAS ver. 9.4 (SAS Institute Inc., Cary, NC, USA) was used for the statistical analysis. According to Korea Centers for Disease Control and Prevention Guideline on KNHANES Raw Data Use, sampling weight was used to analyze the complex survey samples.

First, a chi-square test was used to calculate frequency and percentage (%), which assisted in comparing the differences in WC by the general characteristics and health behaviors of the participants. If the general and health-behavior characteristics were continuous variables, a general linear model was performed. In addition, the average and standard error for the different WCs were calculated.

Second, a logistic regression was performed to examine the impacts of recreational PA on the WC risk level. The impacts of recreational PA on WC were first examined without any adjustments (Model 1). The association between recreational PA and WC was then examined after adjusting the factors of the general characteristics that had a significant relationship with WC (Model 2). The impacts of recreational PA on WC were examined after adjusting the additional factors that had a significant relationship with health behaviors (excluding PA) and WC (Model 3). Lastly, the impacts of recreational PA on WC were examined after adjusting the work PA and transport PA (Model 4). All analysis was performed after categorizing all participants into male/female groups.

## 3. Results

### 3.1. Comparison of Physical Activity by General Characteristics

The average age of the male no-risk group (WC < 90 cm) was 39.62 years, and that of the male at-risk group (WC ≥ 90 cm) was 39.84 years. There was no statistically significant difference between the average ages of the male and female groups. However, the average age of the women in the no-risk group (WC < 85 cm) was 41.32 years, while that of the women in the at-risk group (WC ≥ 85 cm) was 45.09 years. Therefore, the age of the women in the at-risk group was significantly higher than that of those in the no-risk group.

Regarding marital status, although the male married (living apart) group accounted for the largest percentage of the WC at-risk group (55.5%), there was no statistically significant difference. However, a statistically significant difference was found between the different marital statuses of the female participants. The women who were married (living together) accounted for the highest percentage (65.4%), followed by the married (living apart) (62.6%) and single groups (49.5%).

Regarding household income, there were no statistically significant differences in the WC risk level according to household income between men and women.

Employment did not make a significant difference in the WC men at-risk group. However, the percentage of unemployed women in the WC at-risk group (67.5%) was significantly higher than that of employed women (58.4%).

Although there were more men with university/college as their highest education in the WC at-risk group, there was no significant difference between the different educational levels of men who participated. A statistically significant difference was found between the different educational levels among the women as follows: elementary school (72.9%), junior high school (76.6%), high school (60.1%), and university/college (58.6%). This finding indicates that the percentage of the WC at-risk group decreased as the level of education increased.

Regarding menopause, 59% of the premenopausal women were in the WC risk group, while 68.3% of the menopausal women were in the WC risk group. Therefore, the percentage of menopausal women in the WC at-risk group was higher than that of the premenopausal participants (Table 1).

### 3.2. Comparison of Health Behaviors According to Waist Circumferences

Regarding smoking, the percentage of men and women who currently smoke in the WC at-risk group was high; however, this was not statistically significant.

Regarding drinking, the percentage of the WC at-risk group was high in the drinker group for men and the non-drinker group for women without statistical significance.

Regarding hours of sleep, the men with 7–8 h of sleep formed the larger proportion of the WC at-risk group (64.8%), and the women with <7 h of sleep formed the larger percentage of the WC at-risk group (63.6%) without statistical significance.

Regarding energy intake level, the men with adequate energy intake formed a high percentage of the WC at-risk group without statistical significance. The women with adequate energy intake (66.9%) form a significantly higher proportion of the WC at-risk group than those with inadequate energy intake (59.6%).

Regarding work PA, men with >600 METS/week of PA formed the lowest proportion in the WC at-risk group without statistical significance. The women with no work PA comprised the lowest percentage of the WC at-risk group without statistical significance.

In recreational PA, 66.4% of men with “NO” recreational PA, 68.8% of men with <600 Mets/week recreational PA, and 56.2% of men with ≥600 Mets/week recreational PA were part of the WC at-risk group. A percentage of 66.2 of women with “NO” recreational PA, 62.3% of women with <600 Mets/week recreational PA and 50.6% of women with ≥600 Mets/week recreational PA form part of the WC at-risk group. In both men and women, the groups with ≥600 Mets/week recreational PA formed the lowest percentage of WC at-risk group without statistical significance.

Regarding transport PA, men, and women with “NO” transport PA formed the lowest percentage of the WC at-risk group without statistical significance.

Among the men, the percentage of the WC at-risk group was highest in those with sedentary behavior of ≥480 min (67.3%), followed by those with sedentary behavior of <480 min (61.6%) and <240 min (53.5%). There were significant differences in the percentage of WC at-risk groups according to the duration of sedentary behavior. In contrast, among the women, there was no significant difference in the percentage of WC at-risk groups according to the duration of sedentary behavior (see Table 2).

### 3.3. Risk Analysis Regarding the Impacts of Recreational Physical Activity on Waist Circumference

Regarding PA, the percentage of the groups with work PA ≥ 600 Mets/wk (male 12.9% and female 5.6%) and transport PA (male 3.9% and female 3.9%) was significantly lower than the group with recreational PA ≥ 600 Mets/wk (male 35.7% and female 20%). Therefore, this study aimed to examine the relationship between recreational PA and WC.

**Figure 1:**
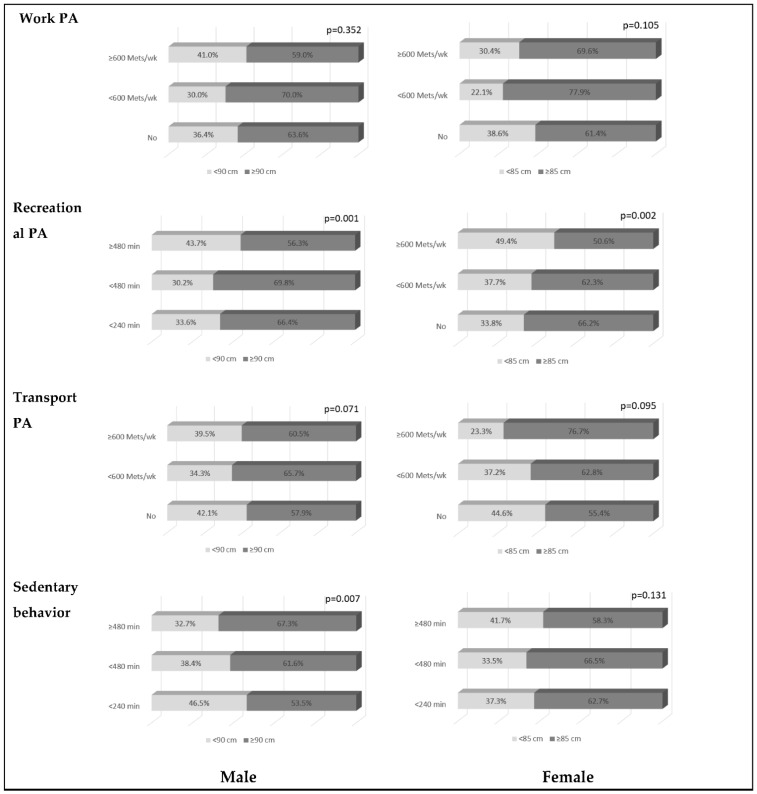
Distribution of WC at-risk group according to the amount of physical activity. Among obese adults, the rate of WC at-risk group according to physical activity level showed a significant difference in recreational PA. Levels of physical activity above 600 Mets/wk showed significantly lower rates of WC at-risk group, and men had significantly higher rates of WC at-risk group as sedentary behavior increased.

There was no statistically significant difference in the odds ratio for WC risk between the group with ≥600 Mets/wk PA and the group with “NO” recreational PA in both the adult male and female groups (Figure 1).

The odds ratio of the male group with ≥600 Mets/wk PA for WC risk was significantly lower than that of the male group with “NO” recreational PA in Model 1 (0.65), Model 3 (0.60), and Model 4 (0.67).

The odds ratio of the female group with ≥600 Mets/wk PA for WC risk was significantly lower than that of the female group with “NO” recreational PA in Model 1 (0.52), Model 3 (0.57), and Model 4 (0.61) (see Table 3).

## 4. Discussion

The worldwide prevalence of obesity has gradually increased [1,23]. Moreover, multi-perspective monitoring is required because of the close association between obesity and chronic disease prevalence [25]. Obesity is generally diagnosed based on BMI [26]. However, some studies have suggested that WC should be considered a factor independent of chronic diseases [27,28]. Furthermore, it is difficult to diagnose obesity using only a single indicator [29,30] because WC may increase independently of BMI or weight [31]; hence, the classification of obesity may differ depending on whether BMI or WC is used [29,32].

Therefore, this study used KNHANES VII (2016–2018) data to examine the impact of the general characteristics, health behaviors, and PA of obese adults (BMI ≥ 25 kg/m^2^) on abdominal obesity and to provide basic data for the improvement and management of abdominal obesity.

First, this study compared the general characteristics of men and women according to Korean abdominal obesity criteria (WC ≥ 90 cm in men and ≥85 cm in women). The percentage of abdominal obesity was high in women who were older, single, unemployed, with low educational attainment, or had attained menopause. However, there were no significant differences between the general characteristics of the male participants.

This finding is consistent with a study identifying factors that influenced BMI in Korean adults using KNHANES, which showed that BMI increases in women as they age. However, another study also reported an association between increased BMI and older age in men and indicated that the risk of obesity was significantly higher in men between the ages of 30 and 39 years [30], which is inconsistent with the findings of this study. These findings suggest that women with obesity have more factors that need to be considered by researchers as general characteristics for the management of abdominal obesity; thus, they require more detailed attention.

In this study, among health-behavior characteristics, the percentage of obese men with WC ≥ 90 cm was significantly higher among those with “NO” PA or PA of <600 Mets/week. In addition, those with WC ≥ 90 cm had a significantly longer sedentary time. In the WC at-risk group, the percentage of women with WC ≥ 85 cm increased when they had “NO” recreational PA, <600 Mets/week of PA, or adequate energy intake. Therefore, the results may indicate that men require management of PA-related factors, whereas women require management of PA and energy intake. This finding is inconsistent with previous findings, which suggest that health-behavior factors influence the BMI of South Korean adults, including high-risk drinking for men and income level and sedentary time for women [30]. Moreover, ≥2 h of watching television affected BMI and abdominal obesity in American adults [33]. However, this study’s findings regarding the association between abdominal obesity in women and energy intake are consistent with the results of another study, which reported that obesity and abdominal obesity increased in American women when they consumed meals prepared away from home [33]. Therefore, the differences in the findings may be attributed to the different target participants, as this study included obese adults only and most other studies included all adults, so their results may be more comprehensive [30,33].

Recommendations for preventing obesity and other diseases include a minimum of 150–300 min/week of moderate-intensity aerobic exercise and 75–150 min/week of high-intensity exercise [4,34]. Despite these recommendations, South Korea reports a consistently inadequate level of PA and has an increasing prevalence of obesity and chronic disease [23,24]. The WHO stated that a minimum of 150 min of PA in individuals with a healthy body weight could prevent various diseases, including obesity [34]. However, the number of studies that independently analyzed the effects of PA on WC in obese individuals remains insufficient.

The current study has some limitations and restricting elements. The cross-sectional study design does not warrant us to draw cause–effect relationships between the obesity and physical activity levels. Additionally, the nature of epidemiological study using surveys with established sets of variables was significantly restrained. For this study, all subjects whose physical activity surveyed by GPAQ was less than 600 Mets/wk and less than 150 min were analyzed as a group that did not engage in physical activity (the elderly group). However, I think it will be meaningful to be able to generalize and analyze data from the National Health and Nutrition Examination Survey, which is representative of the entire population, to a large number of citizens.

This study examined the impact of 600 METs/week of recreational PA on WC after adjusting for the general characteristics that affect WC, health behavior, and work and transport PA. According to the results, <600 METs/week of PA did not result in a statistically significant difference in WC; however, the odds ratio for abdominal obesity decreased to 69% in men and 61% in women when they performed ≥600 Mets/week of recreational PA. Therefore, the performance of ≥600 Mets/week of PA is considered a significant factor in the prevention of WC in adults with obesity. Additionally, low-intensity exercise did not appear to affect WC or BMI. Consequently, performing PA at a certain intensity level is considered necessary to manage abdominal obesity [35]. However, some studies have reported that low-intensity PA has a positive impact on WC, which is inconsistent with the findings of this study [36,37,38,39,40]. Furthermore, the other studies included participants of all age groups, with a minimum age of 18 years or older, whereas this study selected adults with obesity only. Thus, this difference in study participants between studies may have contributed to the inconsistent findings. Therefore, the researchers recommend using the results of this study as basic data for managing abdominal obesity in adults.

## 5. Conclusions

In summary, the factors that affect abdominal obesity in obese Korean adults include general characteristics (such as age, marital status, employment, educational level, and menopause) and health behaviors (recreational PA and energy intake) among women, and health behaviors (recreational PA and sedentary behavior) affect abdominal obesity in men. Additionally, the researchers adjusted for the general characteristics and health behaviors that affected abdominal obesity and examined the changes in the prevalence rate of abdominal obesity according to different levels of recreational PA. The results showed that ≥600 Mets/week of recreational PA decreased the prevalence of abdominal obesity in men and women. Therefore, the general characteristics and health behaviors of men and women should be considered in preventing or treating abdominal obesity in Korean adults and performing ≥600 Mets/week of PA is highly recommended.

## 6. Limitations

This study was analyzed using the Korean National Health and Nutrition Survey, and because of intensive analysis of the characteristics of Koreans, there will be differences in the results according to race. In addition, the data surveyed by GPAQ were analyzed for physical activity, and the subjects who did not respond did not know how much physical activity they had, and they had no choice but to classify it as “NO” and analyze it.

## Figures and Tables

**Table 1:** Comparison of waist circumferences between men and women according to general characteristics (N(%)).

		Male		Female	
		<90 cm	≥90 cm	<85 cm	≥85 cm
Age	Years (M ± SE) ^1^	39.62 ± 0.68	39.84 ± 0.45	41.32 ± 0.80	45.09 ± 0.61
	*p*	0.786		<0.001	
Marital status	Single	157 (40.3)	230 (59.7)	74 (50.5)	72 (49.5)
	Married (living together)	308 (34.6)	598 (65.4)	261 (34.6)	488 (65.4)
	Married (living apart)	16 (44.5)	26 (55.5)	37 (37.4)	68 (62.6)
	*p*	0.123		0.004	
Household income	Q1	116 (40.5)	178 (59.5)	103 (34.3)	190 (65.7)
	Q2	109 (35.3)	213 (64.7)	97 (36.1)	182 (63.9)
	Q3	133 (38)	233 (62)	97 (41.8)	149 (58.2)
	Q4	123 (34.5)	228 (65.5)	75 (42.9)	106 (57.1)
	*p*	0.471		0.221	
Employment	Employed	371 (36.5)	663 (63.5)	219 (41.6)	311 (58.4)
	Unemployed	69 (36.7)	130 (63.3)	125 (32.5)	286 (67.5)
	*p*	0.955		0.019	
Education level	Elementary school	16 (38.4)	24 (61.6)	32 (27.1)	92 (72.9)
	Junior high school	25 (42.9)	34 (57.1)	25 (23.4)	71 (76.6)
	High school	165 (39.7)	270 (60.3)	151 (39.9)	224 (60.1)
	University	237 (34.1)	467 (65.9)	136 (41.4)	209 (58.6)
	*p*	0.257		0.007	
Menopause	No	-	-	232 (41)	342 (59)
	Yes	-	-	110 (31.7)	245 (68.3)
	*p*	-	-	0.013	

^1^ Mean (M), Standard error (SE).

**Table 2:** Comparison of waist circumferences between males and females by health behavioral characteristics (N(%)).

		Male		Female	
		<90 cm	≥90 cm	<85 cm	≥85 cm
Smoking	Current smoker	168 (35.6)	315 (64.4)	16 (36.6)	38 (63.4)
	Former smoker	176 (36.9)	312 (63.1)	28 (42.5)	45 (57.5)
	Never smoker	130 (37.8)	220 (62.2)	320 (37.1)	542 (62.9)
	*p*	0.827		0.757	
Drinking	Non-drinker (under once a month)	105 (39.9)	182 (60.1)	175 (35.5)	318 (64.5)
	Drinker (over once a month)	369 (35.8)	665 (64.2)	189 (39.1)	308 (60.9)
	*p*	0.223		0.305	
Hours of sleep	<7 h	194 (38.3)	342 (61.7)	141 (36.4)	250 (63.6)
	7–8 h	150 (35.2)	267 (64.8)	110 (40.8)	173 (59.2)
	≥8 h	99 (35.9)	183 (64.1)	93 (36)	174 (64)
	*p*	0.645		0.517	
Energy intake	Inadequate	276 (37.9)	461 (62.1)	260 (40.4)	405 (59.6)
	Adequate	205 (35.5)	393 (64.5)	112 (33.1)	224 (66.9)
	*p*	0.423		0.044	
Work PA	No	357 (36.4)	647 (63.6)	317 (38.6)	527 (61.4)
	<600 Mets/wk	18 (30)	49 (70)	10 (22.1)	25 (77.9)
	≥600 Mets/wk	67 (41)	98 (59)	16 (30.4)	45 (69.6)
	*p*	0.352		0.105	
Recreational PA	No	185 (33.6)	381 (66.4)	209 (33.8)	405 (66.2)
	<600 Mets/wk	73 (30.2)	167 (69.8)	51 (37.7)	97 (62.3)
	≥600 Mets/wk	184 (43.7)	246 (56.3)	83 (49.4)	95 (50.6)
	*p*	0.001		0.002	
Transport PA	No	150 (42.1)	232 (57.9)	51 (44.6)	75 (55.4)
	<600 Mets/wk	275 (34.3)	536 (65.7)	283 (37.2)	498 (62.8)
	≥600 Mets/wk	18 (39.5)	25 (60.5)	8 (23.3)	24 (76.7)
	*p*	0.071		0.095	
Sedentary behavior	<240 min	84(46.5)	104(53.5)	60(37.3)	106(62.7)
	<480 min	173(38.4)	292(61.6)	134(33.5)	270(66.5)
	≥480 min	186(32.7)	398(67.3)	149(41.7)	219(58.3)
	*p*	0.007		0.131	

**Table 3:** Risk analysis of recreational PA on waist circumference.

	Male		Female	
	None vs. <600 Mets/wk	None vs. ≥600 Mets/wk	None vs. <600 Mets/wk	None vs. ≥600 Mets/wk
Model 1	1.17 (0.82–1.67)	0.65 (0.49–0.87)	0.85 (0.55–1.30)	0.52 (0.36–0.75)
Model 2	-	-	0.92 (0.60–1.43)	0.57 (0.39–0.83)
Model 3	1.09 (0.75–1.57)	0.60 (0.45–0.81)	0.94 (0.61–1.45)	0.57 (0.39–0.84)
Model 4	1.24 (0.84–1.83)	0.67 (0.50–0.90)	1.01 (0.63–1.61)	0.61 (0.40–0.94)

Model 1: No adjustment. Model 2: Adjusted for age, marital status, job, education, and menopause. Model 3: (male Model 2 + sedentary time, female) Model 2 + energy intake level. Model 4: Model 3 + work physical activity, transport physical activity.

## Data Availability

Not applicable.
